# High-resolution elevation models of Larsen B glaciers extracted from 1960s imagery

**DOI:** 10.1038/s41598-024-65081-6

**Published:** 2024-07-08

**Authors:** Ryan North, Timothy T. Barrows

**Affiliations:** 1 School of Earth, Atmospheric and Life Sciences, Environmental Futures Research Centre, University of Wollongong, Wollongong, NSW 2522 Australia; 2 Securing Antarctica’s Environmental Future, University of Wollongong, Wollongong, NSW 2522 Australia; 3 Chronos Radiocarbon Facility, University of New South Wales, Sydney, 2052 Australia

**Keywords:** Antarctica, Photogrammetry, Sea-level rise, Ice shelf collapse, DEM, Geomorphology, Environmental impact, Climate change, Cryospheric science

## Abstract

Accelerated warming since the 1950s has caused dramatic change to ice shelves and outlet glaciers on the Antarctic Peninsula. Long observational records of ice loss in Antarctica are rare but essential to accurately inform mass balance estimates of glaciers. Here, we use aerial images from 1968 to reveal glacier configurations in the Larsen B region. We use structure-from-motion photogrammetry to construct high-resolution (3.2 m at best) elevation models covering up to 91% of Jorum, Crane, Mapple, Melville and Flask Glaciers. The historical elevation models provide glacier geometries decades before the Larsen B Ice Shelf collapse in 2002, allowing the determination of pre-collapse and post-collapse elevation differences. Results confirm that these five tributary glaciers of the former Larsen B Ice Shelf were relatively stable between 1968 and 2001. However, the net surface elevation differences over grounded ice between 1968 and 2021 equate to 35.3 ± 1.2 Gt of ice loss related to dynamic changes after the ice shelf removal. Archived imagery is an underutilised resource in Antarctica and was crucial here to observe glacier geometry in high-resolution decades before significant changes to ice dynamics.

## Introduction

The Antarctic Peninsula is currently one of the fastest warming regions on the Earth with a mean annual air temperature increase of 2.5 °C from the 1950s to 2000s^1,2^ and substantial warming of subsurface waters^3–5^. Since the 1970s, seven out of twelve ice shelves on the Antarctic Peninsula have rapidly collapsed or retreated significantly^6^. One example was the dramatic collapse of the Larsen B Ice Shelf (LBIS) in March 2002^7,8^ which has been attributed to unusually warm temperatures^9^ causing excessive meltwater accumulation and subsequent hydrofracture^10–13^. Post-ice shelf collapse, former ice shelf tributary glaciers accelerate and thin without the backpressure and calving protection from the ice shelf^14–17^.

Several studies have monitored elevation change, mass loss and retreat patterns of Larsen B tributary glaciers following the ice shelf collapse using modern satellite data^18–24^. In brief, glaciers that were debuttressed by the LBIS collapse responded quickly to dynamic changes and thinned by up to 38 m within one-year post-collapse^19,20,24^. Substantial mass losses occurred in the decade following, especially on wider glaciers with deeper beds (including Crane and Jorum) where basal and lateral friction did not mitigate the progression of dynamic thinning^18,20–22,24^. Crane Glacier alone lost ~ 13 km^3^ of grounded ice in the period 2001–2009 related to the collapse^18^. Thinning continues upstream in former LBIS tributaries despite sea surface cooling and the development of land-fast sea ice that buttressed glacier outlet flow between 2011 and 2021^9,24,25^. While detailed observations and models of retreat exist for post-collapse changes to these glaciers, information for glacier configurations decades before the removal of the LBIS is sparse.

Quantifying the ice loss and contribution to sea-level rise over multi-decadal timescales is important for climate-sensitive areas like the Antarctic Peninsula^26,27^. However, long observational timeseries are rare due to difficult field conditions and persistent cloud cover that interferes with satellite measurements. As a result, mass balance estimates on the Antarctic Peninsula can be relatively short^28–30^ and vary widely (4–42 Gt a^−1^)^31–35^. Recent reviews of global ice volume change note the lack of glacier configuration data for before the 1990s when high-quality surface elevation data was unavailable^26,36^.

Historical images can extend the observational record of glaciers by decades. More than 300,000 historical aerial images of Antarctica were acquired by U.S. Navy cartographers from 1946 to 2000 and are freely available at the Polar Geospatial Centre, University of Minnesota. This wealth of data is underutilised; only a few studies Antarctica-wide employ images like these to reconstruct historical glacier configurations^28,37–40^. Surface elevations can be accurately modelled from historical imagery using structure-from-motion (SfM) photogrammetry^28,41,42^. The technique creates digital elevation models (DEMs) by constructing 3D point clouds of matching features in overlapping photos without the need for the original camera positions or orientations^43,44^.

Here, we apply SfM photogrammetry to 871 aerial images from 1968 to construct high-resolution historical DEMs and orthophotos for the Larsen B region which is data poor at the multi-decadal scale. We use the historical DEMs to precisely measure the net change in surface elevations of Larsen B tributary glaciers (Jorum, Crane, Mapple, Melville, and Flask) between 1968 and 2021. For the same glaciers, we also calculate the surface elevation differences between 1968 and 2001, while they were still buttressed by the LBIS, using the only available DEM from just prior to the ice shelf collapse. Using precise elevation differences, we provide new mass balance and sea-level contribution estimates spanning 53-years and discuss these measurements in the context of existing pre-collapse and post-collapse literature.

## Results

### Glacier geometry in 1968

**Figure 1:**
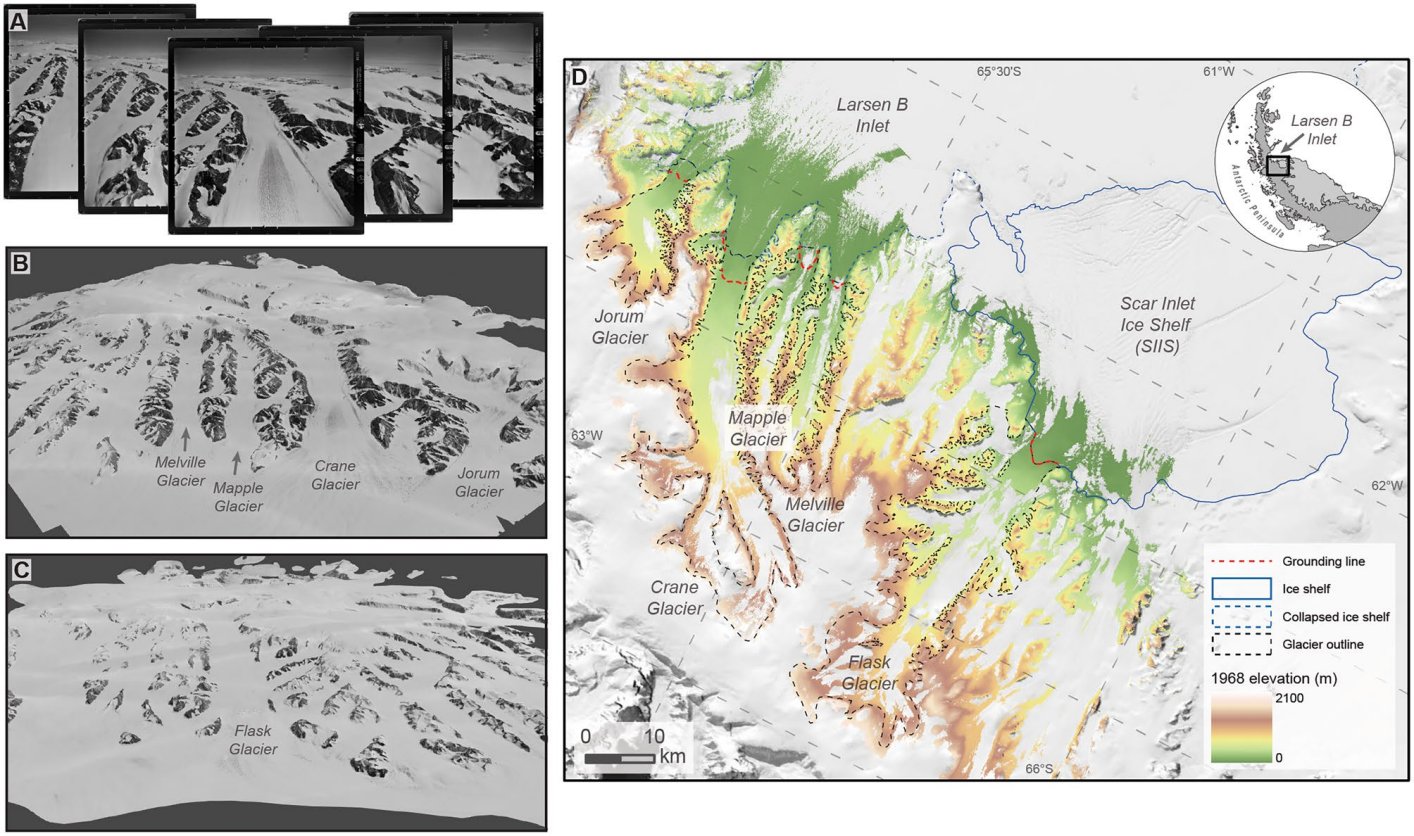
(**A**) Example of overlapping historical aerial imagery (oblique) from December 1968 looking upstream Crane Glacier, (**B,C**) three-dimensional models of the Crane Glacier area and Flask Glacier derived from historical imagery, and (**D**) Glacier outlines (manually delineated to 2021 terminus, see “Methods and data” section) and lines used to separate grounded and floating ice^45,46^ superposed on photogrammetrically derived DEMs representing 1968 surfaces. Grounding lines represent 1999 positions, except for Crane Glacier which represents 2018^45,46^. Inset shows study region in context of the Antarctic Peninsula. Ice shelf outlines are from the SCAR Antarctic Digital Database.

Two DEMs representing glacier surfaces in 1968 for five Larsen B tributary glaciers were created using historical imagery and cover an area ~ 4200 km^2^ (Fig. 1). The DEM that covers the Jorum, Crane, Mapple and Melville Glaciers (i.e., ‘Crane Area’ DEM) is high resolution (3.2 m) and covers 62–91% (mean 70%) of the total surface of these glaciers. Voids in the DEM were produced in areas of minimal surface contrast such as snowfields in the upper catchment. The Flask DEM has a resolution of 13.3 m but covers less of the glacier surface (58%) due to a lack of surface contrast and sparse photo coverage. In both historical DEMs, mountain terrain is well represented and exhibits fewer elevation artefacts than in any individual Reference Elevation Model of Antarctica (REMA) or ASTER DEM.

**Figure 2:**
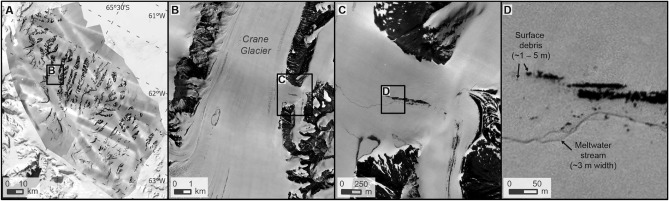
Details of high spatial resolution (1.6 m pixel size) historical orthophoto mosaic covering the Larsen B region in December 1968. (**A**) Full extent of the orthophoto mosaic, (**B**) enlarged region on Crane Glacier, (**C**) a tributary of Crane Glacier enlarged further, and (**D**) metre-scale surface debris and meltwater channels visible in the same tributary of Crane Glacier.

High quality orthophotos of the five glaciers in 1968 created with the same historical imagery are presented in Fig. 2. The orthophoto mosaic covering glaciers in the Crane Area and Flask Glacier are very high resolution with 1.6 m cell sizes. Crevasses, meltwater ponds, meltwater streams and surface debris are visible at the metre scale (Fig. 2). Occasionally, blurring of the orthophotos occurs due to image stretching over areas of poor coverage. Highlights along image acquisition paths arise from variable exposure between oblique and nadir images but the effects of this are only cosmetic.

### Pre-collapse surface elevation differences (1968–2001)

**Figure 3:**
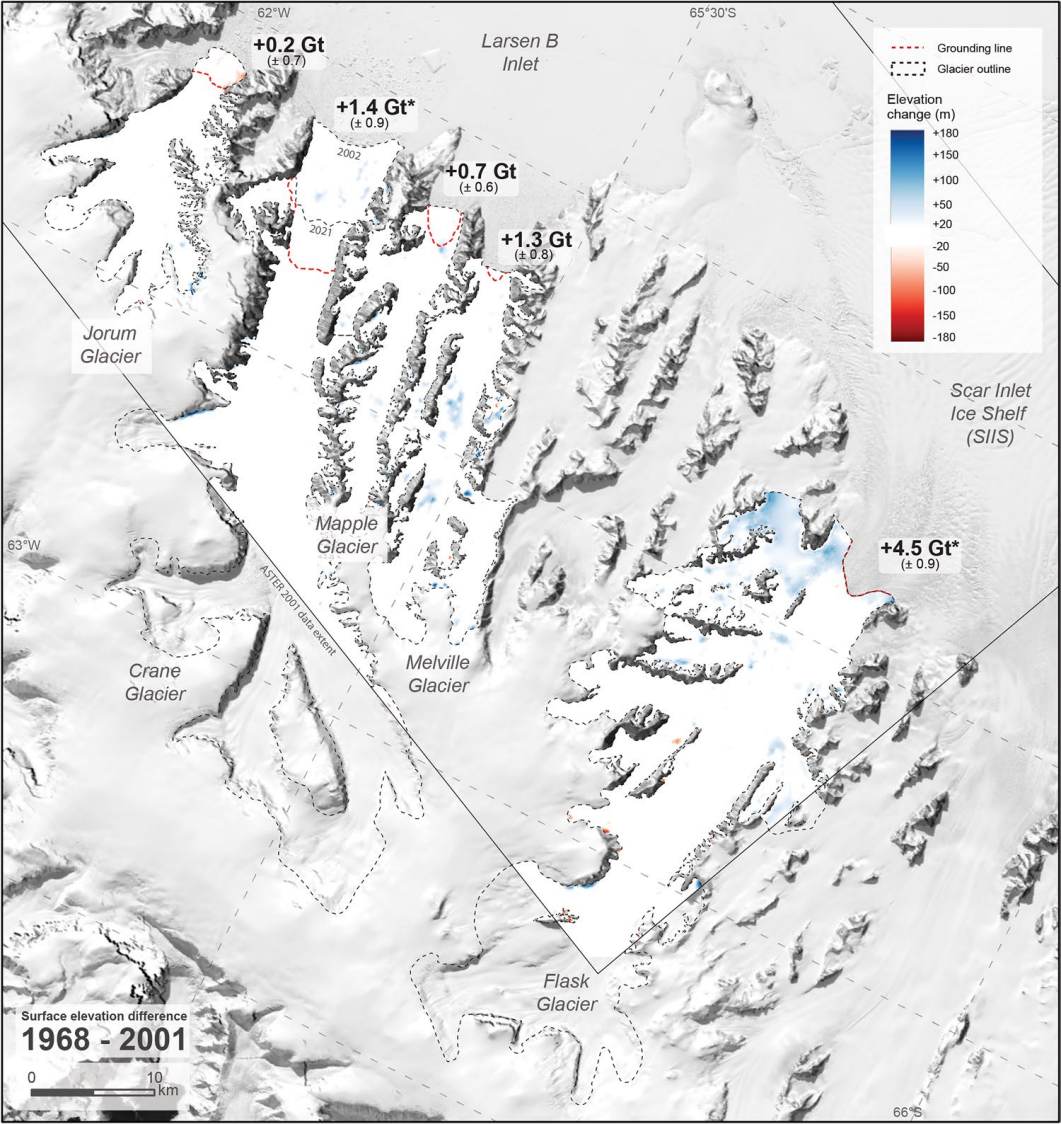
Surface elevation change 1968–2001 for Larsen B tributary glaciers with mass balance labelled at the mouth. Almost all areas exhibit no detectable surface elevation change. Glacier outlines (manually delineated, see “Methods and data” section) and differenced DEMs are shown to 2021 extent except for Crane where the 2002 extent is also shown. All calculations here and in Table 1 are measured on grounded ice upstream of the red dotted line. *Denotes mass balance estimates from less than the entire glacier area.

Between December 1968 and November 2001, the average surface elevations of Larsen B tributary glaciers did not change beyond the limits of detection or had slightly increased (Table 1, Fig. 3). This correlates to minor increase in volume when detectable. Jorum Glacier is the only glacier that showed minor thinning near the grounding zone, otherwise the spatial pattern of surface elevation change during this period is uniform. Non-systematic, isolated patches of apparent thickening and thinning remain in the 1968–2001 differenced DEMs despite smoothing of noisy data in the ASTER 2001 DEM. This inherited noise does not affect the overall trend of undetectable change to minor thickening more than the uncertainty of the elevation data. For Crane and Flask Glaciers, the surface elevation change cannot be observed in the upper catchment due to the limited coverage of the 2001 ASTER DEM.

**Table 1:** Glacier surface elevation and volume change between 1968–2001 (pre-collapse), 2001–2021, and 1968–2021 (net) over the measured grounded ice area.

	Measurement area	Surface elevation change	Volume change		
	(km^2^)	Mean ± 2σ (m)	(km^3^)	Mass equivalent ± 2σ (Gt)	Sea-level rise equivalent (mm)
1968–2001					
Jorum	133.7 ± 0.9	1.4 ± 5.6	0.2 ± 0.8	0.2 ± 0.7	0.000 ± 0.002
Crane*	242.1 ± 1.1	6.2 ± 4.1	1.5 ± 1.0	1.4 ± 0.9	− 0.004 ± 0.003
Mapple	110.7 ± 0.9	6.5 ± 6.1	0.7 ± 0.7	0.7 ± 0.6	− 0.002 ± 0.002
Melville	189.6 ± 1.2	7.4 ± 4.7	1.4 ± 0.9	1.3 ± 0.8	− 0.004 ± 0.002
Flask*	461.1 ± 1.9	10.6 ± 2.1	4.9 ± 1.0	4.5 ± 0.9	− 0.012 ± 0.003
Total*			8.7 ± 1.9	8.1 ± 1.8	− 0.022 ± 0.005
2001–2021					
Jorum	133.7 ± 0.9	− 36.4 ± 4.9	− 4.9 ± 0.7	− 4.5 ± 0.6	0.012 ± 0.002
Crane*	242.1 ± 1.1	− 89.9 ± 3.6	− 21.8 ± 0.9	− 20.0 ± 0.8	0.055 ± 0.002
Mapple	110.7 ± 0.9	− 11.4 ± 5.4	− 1.3 ± 0.6	− 1.2 ± 0.6	0.003 ± 0.002
Melville	189.6 ± 1.2	− 12.4 ± 4.1	− 2.3 ± 0.8	− 2.1 ± 0.7	0.006 ± 0.002
Flask*	461.1 ± 1.9	− 14.8 ± 2.6	− 6.8 ± 1.2	− 6.3 ± 1.1	0.017 ± 0.003
Total*			− 37.1 ± 1.9	− 34.1 ± 1.7	0.093 ± 0.005
Net 1968–2021					
Jorum	133.7 ± 0.9	− 35.0 ± 3.3	− 4.7 ± 0.5	− 4.3 ± 0.4	0.012 ± 0.001
Crane	518.7 ± 1.6	− 58.1 ± 1.7	− 30.2 ± 0.9	− 27.7 ± 0.8	0.076 ± 0.002
Mapple	110.7 ± 0.9	− 5.8 ± 3.6	− 0.6 ± 0.4	− 0.6 ± 0.4	0.002 ± 0.001
Melville	189.6 ± 1.2	− 5.6 ± 3.6	− 1.1 ± 0.5	− 1.0 ± 0.5	0.003 ± 0.001
Flask	635.1 ± 2.2	− 2.9 ± 0.8	− 1.8 ± 0.6	− 1.7 ± 0.5	0.005 ± 0.001
Total			38.4 ± 1.4	35.3 ± 1.2	0.098 ± 0.003

**Figure 4:**
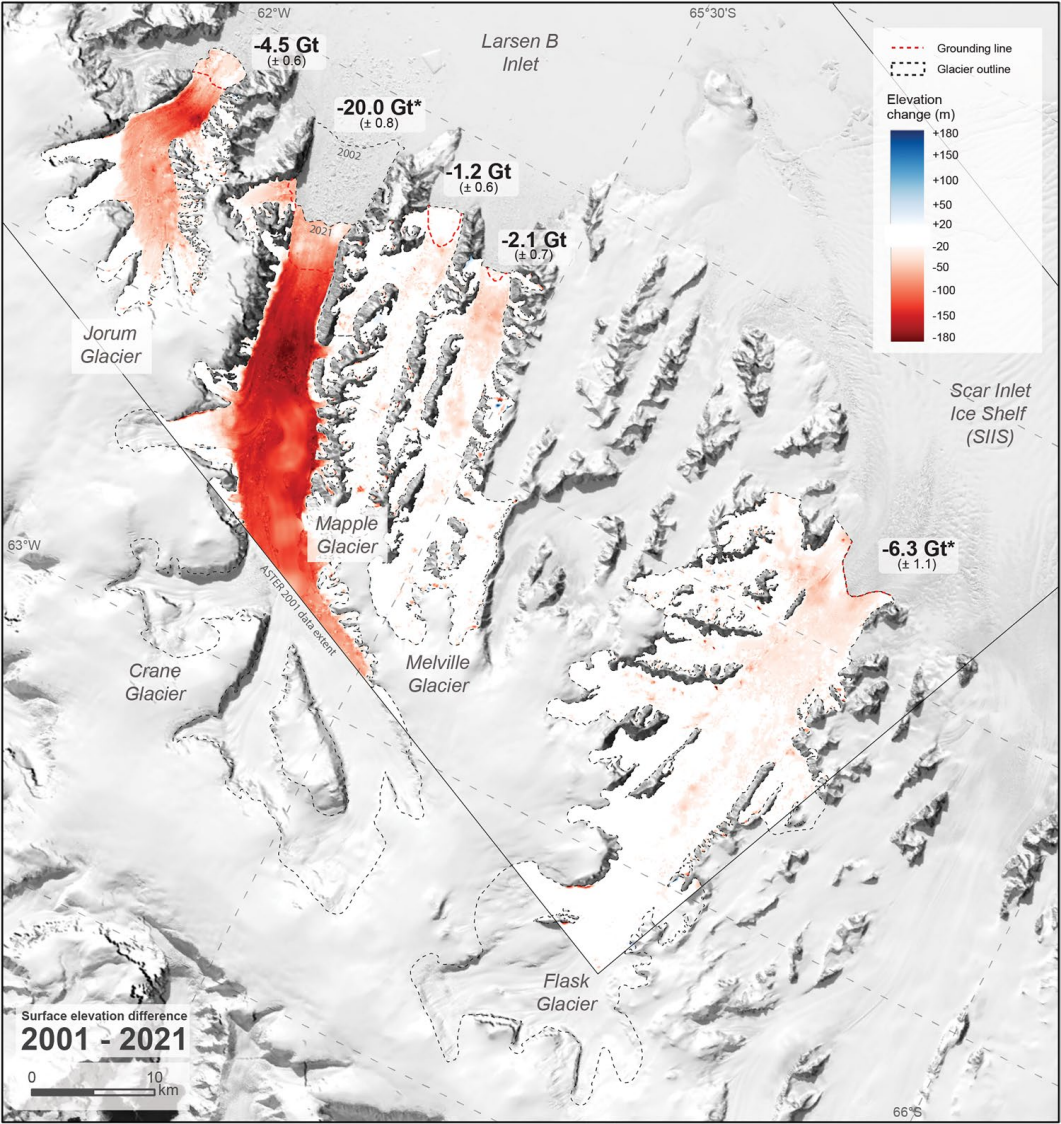
Surface elevation change 2001–2021 for Larsen B tributary glaciers with mass balance labelled at the mouth. All glaciers exhibit surface lowering at least near the grounding line, and Crane and Jorum Glaciers exhibit significant lowering further upstream. Glacier outlines (manually delineated, see “Methods and data” section) and differenced DEMs are shown to 2021 extent. All calculations here and in Table 1 are measured on grounded ice upstream of the red dotted line. *Denotes mass balance estimates from less than the entire glacier area.

*Reported numbers do not encompass entire glacier area due to limited 2001 ASTER DEM extent, see Figs. 3 and 4.

### Post-collapse surface elevation differences (2001–2021)

Over two decades between November 2001 and January 2021, all glaciers exhibited net surface lowering in at least the ablation zone (Fig. 4). In the region where data is available for 2001, surface elevations on Crane Glacier lowered by a mean of − 89.9 ± 3.6 m but up to 186 ± 23 m in a localised area ~ 6.5 km upstream of the grounding line. Jorum Glacier lowered by up to 165 ± 23 m in the ablation zone but also > 30 m in upstream on the main trunk. Thinning was limited above tributary junctions on Jorum Glacier. Mapple Glacier surface elevations lowered near the grounding line by 20–30 m but change was less than detection limits for upstream regions. Similarly, Melville Glacier lowered by 40–50 m near the grounding line but changed little upstream. Flask Glacier thinned by 40–50 m (max 87 ± 23 m) for most of the area near the grounding line but exhibited no detectable change where data is available upstream (Fig. 4). Small, isolated positive or negative anomalies in the 2021–2001 differenced DEM are inherited from noise in the 2001 ASTER DEM but do not affect overall measurements beyond the limits of detection.

### Net magnitude and pattern of thinning (1968–2021)

**Figure 5:**
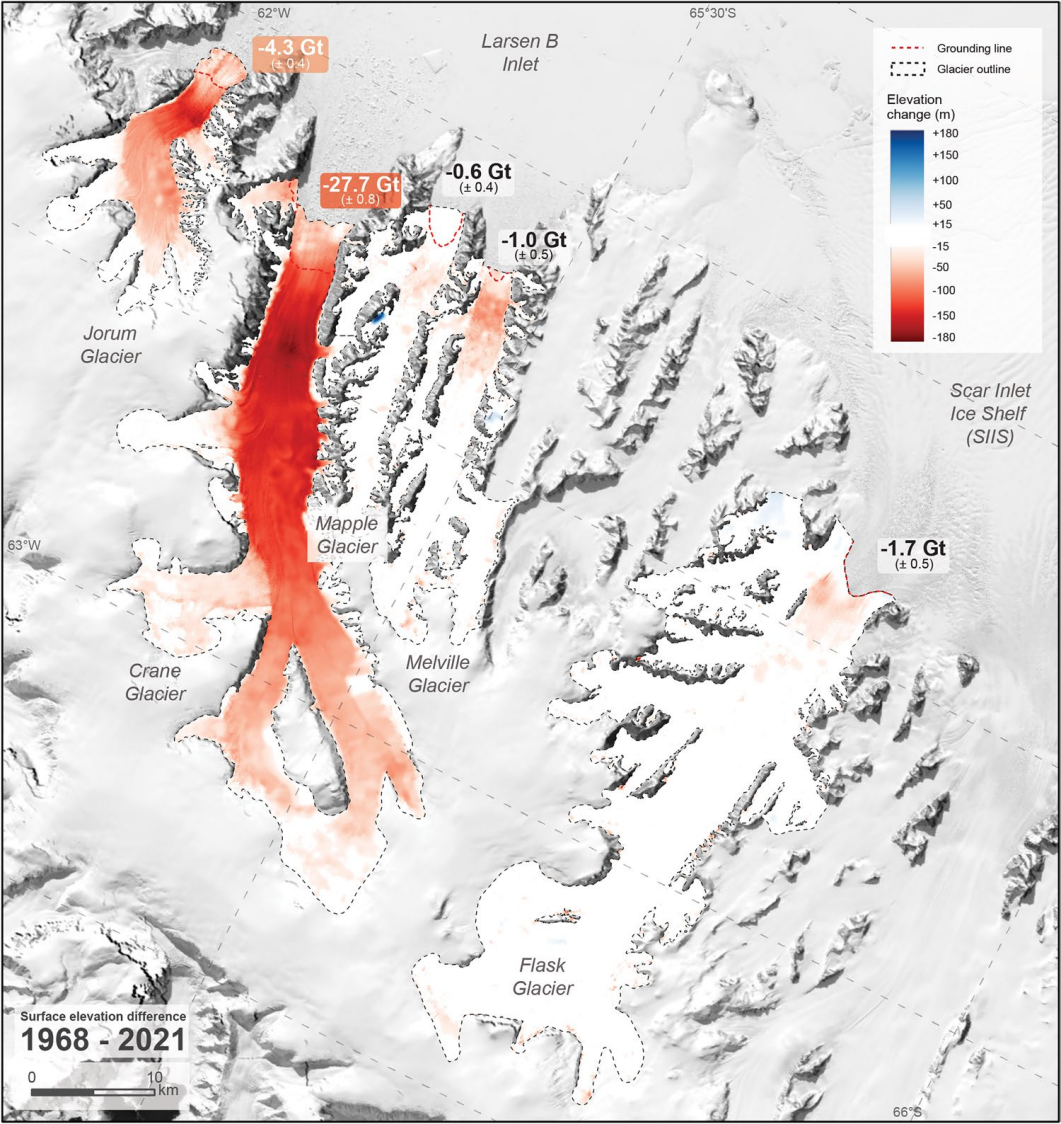
Surface elevation change 1968–2021 for Larsen B tributary glaciers with net mass balance labelled at the mouth. Glacier outlines (manually delineated, see “Methods and data” section) and differenced DEMs are shown to 2021 extent, but all calculations here and in Table 1 are measured on grounded ice upstream of the red dotted line.

All glaciers exhibited surface lowering over the period 1968–2021 that is of similar magnitude and pattern to the 2001–2021 differences (Table 1, Figs. 4, 5). For glaciers now without a buttressing ice shelf, the greatest magnitude and area of thinning occurred on larger, wider glaciers (Jorum and Crane) compared to the smaller, narrower glaciers (Mapple and Melville). Crane Glacier exhibits the most significant net elevation loss of up to 181 ± 15 m in the ablation zone and > 60 m of elevation loss in the accumulation zone. On Jorum Glacier, net thinning of up to 145 ± 15 m occurred in the ablation zone and thinning of 20–30 m occurred upstream. The Mapple and Melville Glaciers thinned up to 31 ± 15 m and 49 ± 15 m near their termini. Flask Glacier, which remains buttressed by the Scar Inlet Ice Shelf (SIIS), showed little detectable change for most its area but did exhibit 40–50 m of thinning near the grounding zone. Notably, thinning continues but is limited above tributary junctions upstream on Crane Glacier.

### Net grounded ice loss (1968–2021)

The volume loss of grounded ice for two periods (2001–2021 and 1968–2021) was derived using the difference in surface elevations over the area upstream of the grounding zone (Table 1). Where entire glacier areas were covered by DEMs, the volume difference 2001–2021 is slightly larger than the total difference 1968–2021 but agree within error. This implies that for glaciers not fully covered by the 2001 DEM extent, the net 1968–2021 grounded ice loss approximates the magnitude of post-collapse change. The glaciers that suffered large surface elevation losses far upstream (Jorum and Crane) lost the most volume of grounded ice. Volume loss on Flask Glacier exceeded the losses on the Mapple and Melville Glaciers due to the greater magnitude of lowering over a similar total area of lowering, despite the area being small relative to the size of Flask Glacier (Fig. 5). Volume loss converted to sea-level rise equivalent shows that the net sea-level contribution 1968–2021 from Crane Glacier (0.076 mm, with an uncertainty of ± 0.002 mm arising from the uncertainty of the volume change) greatly outweighs the contribution from the other glaciers combined (Table 1).

Using estimates of the original volume, which vary substantially due to large uncertainties in bedrock elevations in this region^46^, Jorum Glacier lost the most ice volume relative to its size in 1968 (10–37%). Crane Glacier lost 10–20% of its ice volume, followed by the Mapple (2–10%) and Melville (2–5%) Glaciers. Flask Glacier decreased by < 1%. The original volume was calculated by differencing 1968 surface elevations from two different bedrock elevation models^47,48^ and multiplying by the grounded ice area. The lower estimates of proportional change were generated by using a deeper bedrock model^47^; upper estimates were generated by using a shallower bedrock model^48^.

### Validating surface elevation change using satellite imagery (1963–2021)

**Figure 6:**
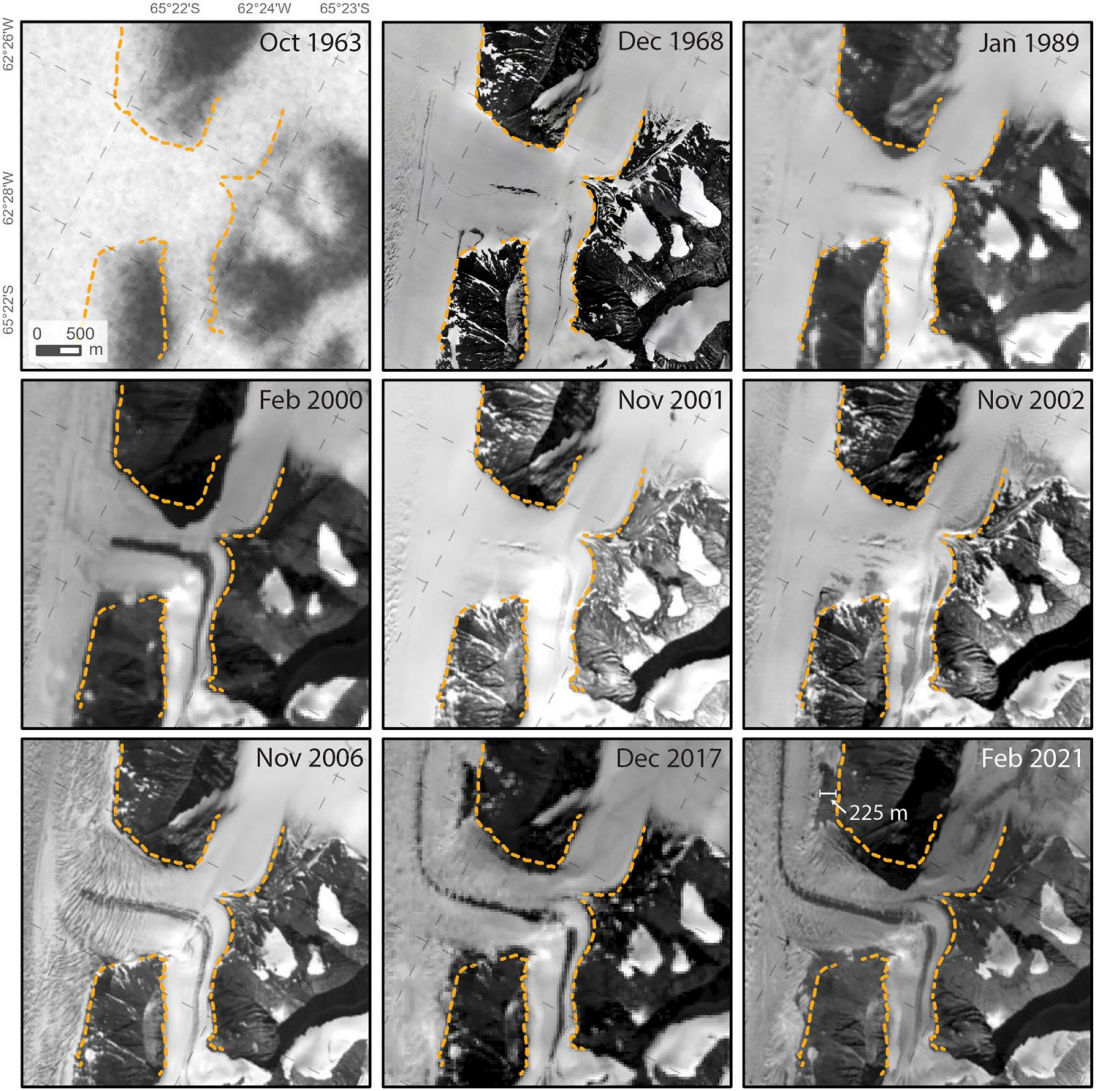
Optical image timeseries 1963–2021 of a tributary junction on Crane Glacier (same as area in Fig. 2C) depicting the expansion of ice-free areas over time. The largest difference between 1963 and 2021 along the valley wall of Crane Glacier’s main trunk is ~ 225 m. Orange dotted line represents the pre-collapse ice margin delineated from the high-resolution orthophoto from 1968. Scale and coordinates in the 1963 panel applies to all panels.

Individual cloud-free images of the Larsen B area cannot directly provide elevation data but can reveal changes to ice margins along valley walls indicative of thinning. Between 1963 and 1989, declassified intelligence satellite photography, historical aerial imagery and Landsat imagery show that ice margins at a representative tributary junction downstream on Crane Glacier did not change (Fig. 6). In 2000, ice-free areas expanded slightly, and a local medial moraine is more exposed relative to 1989. However, in November 2001 the ice margins returned to 1960s–1980s positions, until November 2002 (post-collapse) when ice margins retreated to 2000 positions. After 2002, ice margins gradually retreated to 2021 (Fig. 6).

A net lateral retreat between 1963 and 2022 of Crane Glacier ice margins by ~ 225 m away from valley walls sloping ~ 37° coarsely equates to ~ 170 m of vertical ice thinning, in line with net elevation losses measured by differencing DEMs over the period 1968–2021 (Fig. 5). With only a limited number of images available to monitor glacier margins in the pre-collapse period, it remains ambiguous whether the images are representative of long term (multi-year to decadal) trends or short term (event-scale) fluctuations. Evidence that short-term, event scale fluctuations may affect scenes is visible by the difference in snow accumulation and/or retention in bedrock fractures and glacier crevasses between 1968 and 1989, or 2000 and 2001 (Fig. 6).

## Discussion

### Photogrammetry of historical imagery on the Antarctic Peninsula

This study presents the first precise and high-resolution historical DEM of glaciers bordering the former LBIS. The DEM adds to the small existing collection of historical elevation data Antarctica-wide^28,37–40^. In addition to extending the temporal record of glacier change, the main advantage of our products is the high spatial resolution (3.2 m and 13.3 m for DEMs; 1.5 m and 6.6 m for orthophotos) which compares to the resolution of modern-day satellite derived DEMs. The other advantage is the high precision of the DEMs (Table 2). The precision of the differenced DEMs is as good as 15.2 m, and no greater than 21.5 m. Our historical products are almost twice as precise and substantially higher resolution than previous historical products in the area^38^ (cf. standard deviation ± 42.87 m, spatial resolution 500 m) which makes the ~ 56-year-old data useful for detailed glacier change analysis.

**Table 2:** Co-registration parameters and difference statistics over stable terrain for differenced DEMs.

DEM difference	Master DEM	Co-registration parameters (m)			Stable terrain statistics (m)	
		dx	dy	dz	Mean	SD
2001–1968 (Crane area)	1968	24.07	17.03	26.50	− 4.16	18.74
2001–1968 (Flask area)	1968	27.25	20.63	18.98	− 9.01	21.50
2021–2001 (all)	2021	21.33	16.08	14.89	14.18	22.81
2021–1968 (Crane area)	2021	− 4.05	1.49	− 8.16	6.40	15.22
2021–1968 (Flask area)	2021	− 4.92	− 1.23	1.90	− 0.06	19.21
1968 (Crane area)—ICESat-2	ICESat-2	− 7.17	1.91	− 8.36	− 6.73	24.63
1968 (Flask area)—ICESat-2	ICESat-2	− 4.48	− 4.43	− 0.55	− 0.67	21.61
2001—ICESat-2	ICESat-2	14.42	13.45	17.05	− 2.07	25.79
2021—ICESat-2	ICESat-2	0.50	− 0.60	0.07	− 0.76	4.99
Vector sum						
1968 (Crane area)/2001/ICESat-2		7.25	15.36	8.69		
1968 (Flask area)/2001/ICESat-2		9.94	9.02	16.50		
2021/2001/ICESat-2		14.92	12.85	17.12		
1968 (Crane area)/2021/ICESat-2		− 6.67	1.31	− 8.29		
1968 (Flask area)/2021/ICESat-2		− 3.98	− 5.03	− 0.48		

Three studies have extracted surface elevation data from historical imagery from as early as 1947 for small glaciers on the western Antarctic Peninsula^37,39,40^. These studies used traditional digital photogrammetry and obtained very precise surface elevation measurements with root mean square errors of 2–15 m but were limited by the labour of creating high-resolution data for larger areas. By using modern SfM-photogrammetry software in our study we were able to create high-resolution, precise DEMs using 871 photos to cover an area of ~ 4200 km^2^ with relative ease and rapidity. The manual labour involved during GCP placement and validating image matches is still reasonable and cost effective. In a study of Byrd Glacier in East Antarctica using images from 1978, it was shown that using only three well-positioned GCPs can provide precise, high-resolution surface elevations^28^.

### Overall stability of Larsen B tributary glaciers 1968–2001

Historical DEMs allow glacier geometry to be observed in detail decades before high resolution satellite surface elevation data were available. By using the historical DEMs created here, we show that surface elevations on Larsen B tributary glaciers were relatively unchanged or increased slightly between December 1968 and November 2001, just two months prior to the ice shelf collapse (Fig. 3). Ice margins visible in the limited available pre-collapse imagery also suggest glacier stability between at least 1963 and 1989 (Fig. 6). However, expansion of ice-free areas in satellite imagery from 2000 suggests some surface lowering during the 1990s. This is unquantifiable without elevation data or cloud-free satellite imagery, but is plausible considering warm air and sea surface temperature anomalies and strong surface melt have been recorded and associated with La Niña and positive Southern Annular Mode patterns in the late 1990s^9,49^. Record snowfall in 2001 in the area^49^ means that the 2001 elevation data represents glacier surfaces higher than other years immediately before the LBIS collapse, and therefore the net difference 1968–2001 does not capture the strong surface melting and low precipitation in the 1990s^49^. The temporary increase in accumulation in 2001 can also be observed by comparing ice margin positions to the year before (2000) when surface melt rates were significantly higher, and the year after (2002) when surface melt rates were high and the LBIS had been removed (Fig. 6).

Despite being less precise and coarsely resolved, surface elevations of the same area in 1963 also show no significant change prior to the LBIS collapse^38^. No other pre-collapse elevation data for these glaciers exist, although unchanging glacier velocities affirm the stability of these glaciers between 1995–2000^14,19,50^. Hence, surface elevations of the glaciers analysed here were relatively stable prior to 2002 but potentially lowered leading up to the LBIS collapse. Relatively minor short-term fluctuations in glacier surface elevations pre-collapse were controlled by surface melt and accumulation patterns rather than dynamic forcing from changes at the mouth. Decades of overall stability in glacier surface elevations while local air and sea surface temperatures fluctuate^9,49^, and the regional average temperature increases by several degrees^1,2^, highlights the importance of ice shelves in buttressing ice flow^15^.

By comparison, tidewater glaciers that were not buttressed by an ice shelf on the western Antarctic Peninsula were thinning over a similar period. Surface elevations lowered by 20–30 m on average (maximum 50 m) on twelve small (~ 70 km^2^) tidewater glaciers over different decadal-scale periods during 1947–2010^39^. Differences between 1956 and 2014 surface elevations on another sixteen small tidewater glaciers along the western Antarctic Peninsula revealed a combined mass loss of − 1.862 ± 0.006 Gt^37^. Unlike Larsen B tributary glaciers where a buttressing ice shelf largely controlled glacier dynamics, mass loss from tidewater glaciers on the western Peninsula is generally correlated with atmospheric forcing^39^. However, the spatial pattern of glacier change is variable on the western Peninsula and for dynamically controlled Larsen B tributary glaciers (Figs. 3, 4, 5) which makes generalising driving factors across the Peninsula a non-trivial task^22,37^.

### Thinning of Larsen B tributary glaciers post-ice shelf collapse

The majority of the net difference we calculate between 1968 and 2021 comprises of post-ice shelf collapse change to 2021. This is exhibited by the similar magnitude and pattern of thinning for the available data in 2001–2021 and 1968–2021 differenced DEMs (Figs. 4, 5). Net differences between 1968 and 2021 slightly underrepresent the 2001–2021 surface elevation change due to minor elevation increases between 1968 and 2001 (Table 1). Note that data from 2001 only covers the lower reaches of Crane and Flask Glaciers, so the mean surface elevation change and derived volume change predominantly represents change to the ablation area. These statistics are not comparable to statistics from the entire glacier, so it is assumed that the net 1968–2021 elevation differences represent the post-collapse change for Crane and Flask Glaciers.

There is extensive literature discussing post-collapse changes to Larsen B tributary glaciers which we can use to contextualise our measurements^18–20,22,24,46,51,52^. Crane Glacier exhibits the most dramatic change between glaciers in this study and demonstrates the rapid changes in glacier dynamics that can occur following an ice shelf breakup, including the propagation of thinning into the interior and tributaries^18,22,24,51^ and rerouting of subglacial hydrology^52^. Reduced surface elevation losses near the 2021 termini of Crane Glacier are likely due to compensation by buoyant floating ice^18^ and/or thickening of the ablation zone over the last decade due to the formation of a buttressing ice tongue and land-fast sea ice^24,46^. The disintegration of land-fast sea ice in 2022 will perturb the glacier terminus position and thickness in the future^25^.

The smaller Jorum Glacier also exhibits significant change and continues to thin; results here show losses have more than doubled since 2008 and progressed upstream^18^. The Mapple and Melville Glaciers exhibit surface lowering of > 30 m near their termini but high lateral stresses in the narrower valleys regulate the total magnitude and rate of thinning^22,51^. Significant net thinning (up to 50 ± 19 m) in an isolated area near the Flask Glacier grounding zone since 1968 suggests a potential reduction in the buttressing effect of the less resilient SIIS^53,54^. However, most of Flask Glacier’s surface does not exhibit change beyond the detection limits.

Glaciers debuttressed by the LBIS collapse exhibit significant ice drawdown compared to other tidewater glaciers on the Antarctic Peninsula. Surface elevation losses from 1968 to 2010 for 12 tidewater glaciers on the western Antarctic Peninsula average 20–30 m (maximum 50 m) within 1 km of glacier termini^39^. The larger Crane and Jorum Glaciers have thinned ~ 100 to ~ 150 m more than this at glacier fronts and thinned by > 50 m upstream since 1968. Note that annual surface melt rates of Crane Glacier (and other glaciers on the Antarctic Peninsula) are large relative to other Antarctic glaciers^22,55^ but are still dwarfed by the magnitude of drawdown post-ice shelf collapse.

Estimates for the mass balance of grounded ice over the period 1968–2021 for the Jorum, Crane, Mapple, Melville and Flask Glaciers totals − 35.3 ± 1.2 Gt, equating to 0.098 ± 0.002 mm sea-level rise (Table 1). The total mass change rate is − 1.87 ± 0.06 Gt a^−1^ averaged over the period March 2002–January 2021 using net 1968–2021 difference that covers the entire glaciers. Note that ice loss from Crane Glacier accounts for most of the total (27.7 ± 0.8 Gt, or − 1.47 ± 0.04 Gt a^−1^ for the post-collapse period). These numbers are minima and underestimate the actual magnitude ice loss because the ice lost to retreat and melting below the grounding zone was not accounted for here. Even with a conservative mass balance rate averaged over ~ 19 years, Crane Glacier itself contributes disproportionately to regional^31^ (− 24 Gt a^−1^, averaged over 1979–2017 for the Antarctic Peninsula) and global^33^ (− 267 ± 16 Gt a^−1^, averaged over 2000–2019) ice mass balance rates. Mass balance estimates for the Peninsula can vary (6–42 Gt a^−1^) depending on the time period measured^31,56^. For the Larsen A and B areas, estimates of mass balance also vary widely (4–31 Gt a^−1^) and is likely a result of the variable behaviour of individual tidewater glaciers and short observational periods^18,22,32,57^.

Several studies have shown that the thinning of Larsen B tributary glaciers contains shorter periods of variability. Dynamically-driven rapid retreat, thinning and mass loss occurred immediately after the ice shelf collapse to ~ 2005^18–20,52^ including localised drawdown related to disrupted subglacial hydrology in Crane Glacier^52^. Slower retreat and reduced ice drawdown occurred during the period 2005–2010^22,24^ and a minor readvance and thickening of the ablation zone during the period 2010–2021^24,46^. In the last decade, thinning has continued upstream and into tributaries despite minor thickening downstream associated with the persistent negative sea surface temperature anomalies^9^ that formed buttressing land-fast sea ice^25^. Although these changes cannot be temporally resolved by the net 1968–2021 elevation differences, the consequent spatial patterns of drawdown two decades post-collapse are revealed when compared to the pre-collapse baseline (Fig. 5)^18,22,28,54^.

### Outlook and applications of historical DEMs

The availability of high-quality historical imagery of the Larsen B area was critical for creating precise and high-resolution DEMs and orthophotos for such a data poor region. Using the historical DEM in conjunction with the few cloud-free, pre-collapse satellite images, we show in detail that surface elevations on the tributaries of the former LBIS were relatively unchanged between 1968 and 2001. This aligns with other measures of glacier stability pre-ice shelf collapse^14,19,38,58^. We add new measurements of total thinning and grounded ice loss over the multi-decadal scale period 1968–2021 (Table 1) which will be vital for improving confidence in longer-term mass balance models of the Antarctic Peninsula^27^. Our detailed maps of elevation differences between 1968 and 2021 depict the net magnitude and spatial pattern of drawdown and represents previously reported short term responses from Larsen B tributary glaciers^18–20,22,24,46,51^.

The high-resolution historical DEMs created here would be useful for a variety of other applications. For example, the pre-collapse glacier geometry would be useful for initialising numerical ice flow models^46^, glacio-isostatic adjustment models^59^ or subglacial hydrology models^52^. The high-resolution DEMs or orthophotos may also provide another timestep for feature tracking to measure flow velocity^28^. Given the coverage and detail of the mountainous terrain, the historical products could be a useful geomorphological baseline to identify mass movements after modern ice loss^60,61^. Further, the expansion of ice-free areas since 1968 in the Larsen B region could be tracked and used to inform models of ecological succession^62^. Additionally, the high resolution of the orthophotos and DEMs would be advantageous to identify target areas for surface exposure dating to determine ice thickness during the last glacial maximum.

Future applications of available historical imagery should focus on glacier conditions prior to ice shelf breakup on the northern Antarctic Peninsula or extend the record of tributary glaciers of vulnerable ice shelves such as the Scar Inlet Ice Shelf and the Larsen C Ice Shelf^53,63^. If photo coverage is limited, smaller glaciers can be investigated because less photos are required to create a useful DEM of the glacier surface^37,39^. In any case, archived historical images are an invaluable resource to extend the record of glacier change across Antarctica.

## Methods and data

### Historical aerial photographs

Historical trimetrogon (overlapping left oblique, nadir, and right oblique) photographs of the Antarctic Peninsula were acquired from the University of Minnesota’s Polar Geospatial Centre (PGC) archives. Cloud-free photos covering the study area were acquired by the US Navy on 21st, 23rd and 27th December 1968 on large format 9 × 9 in greyscale film and scanned at 1000 dpi by the United States Geological Survey (USGS) Earth Resources Observation and Science (EROS) centre. Of the available photos, 503 were used to construct a single DEM that covered the Jorum, Crane, Mapple and Melville Glaciers and 368 were used for the Flask Glacier’s elevations. Corrections were applied to the scanned photos to reduce errors in the photogrammetric process^64^. Fiducial marks on film borders were used to rectify small translations and rotations induced during scanning before photos were cropped to a common square dimension without film borders. Exposure and contrast were manually and subjectively edited for each photo to improve clarity and feature detection^64^. The photos used in this study are listed in Supplementary Material 1 and their calculated positions are presented in Supplementary Material 2.

### Modern surface elevation data

The Reference Elevation Model of Antarctica version 2 (REMA^65^) was used as a latitude, longitude and elevation reference for exposed bedrock and modern ice surfaces. Several 2 m resolution DEM strips from the 2020/2021 summer were mosaicked to create a reference DEM after masking clouds, water and bad edge data (mask provided by the PGC) and co-registering to ICESat-2 (details in “Co-registration” section). This DEM is assigned the nominal date 1st January 2021. Strips were chosen to cover the whole catchment within the shortest possible time span of acquisition dates so that error from variable ice cover was reduced (Supplementary Material 1). Some voids are present in REMA strips due to cloud cover or shadows, typically surrounding steep nunataks.

The only other relatively high-resolution DEM available prior to the ice shelf collapse was a Level 3 orthorectified ASTER DEM from 22nd November 2001 which we used to measure surface elevation differences before the significant reduction in buttressing forces. The ASTER DEM was obtained from the USGS *EarthExplorer* archives (https://earthexplorer.usgs.gov/). The ASTER DEM was manually masked where clouds caused major elevation artefacts and smoothed using the mean of a moving 7 × 7 px window to reduce noise artefacts. Surface melting, ponding and meltwater drainage can alter the surface Antarctic Peninsula over the timescale of several days^66^ and this variation may be captured across data with different timestamps. The effect of surface melt on the differenced elevation results is negligible compared to the magnitude of change that has occurred post-ice shelf collapse.

### Generation of historical surface elevations and orthophotos

DEMs representing the 1968 surface were created in the SfM photogrammetry software *Agisoft Metashape*. Nadir and oblique images were processed together and images were aligned without a camera calibration model since no suitable model was available for this dataset. Image artefacts that cause false matches between photos (such as text, dust, and plane propellers) were masked before photo alignment, as well as the sky, clouds, and obviously out of focus regions towards the horizon in oblique photos. The alignment process was iterated to create an accurate DEM using photos that have poor overlap (65% along-track, 20% cross-track) compared to modern standards^41^ and imprecise initial camera positions (up to 1000 m error). An initial photo alignment was run that included the manual placement of 15 tie points where photo overlap was limited. Tie point positions were chosen so that at least three tie points were visible in each photo (resulting in > 2600 total individual placements; Supplementary Material 2). The point cloud model was transformed to a real-world three-dimensional surface by adding 20 GCPs (at least three placements per photo, i.e., > 2600 total individual placements; Supplementary Material 2). GCPs were located by manually identifying matching bedrock features in the archival imagery and the reference DEM products. A dense cloud was produced using the imagery at original resolution for the Crane Area DEM or downscaled to ¼ resolution for the Flask DEM (which compromises spatial resolution for increased coverage). The dense cloud was filtered to remove erroneous points originating from minimal image overlap and lack of feature detection on the ice shelf. A DEM was then generated from the filtered dense cloud without interpolation across data voids larger than the grid size. Orthophoto mosaics were also produced using a depth map-derived polygonal mesh as the base surface. Detailed notes of the parameters used during processing in Metashape are provided in Supplementary Material 3.

### Co-registration

Each pair of differenced DEMs were co-registered to each other before change measurements were made^67^ (Table 2). Large corrections (> 20 m in *x*, *y* and *z*) were necessary to correct the ASTER DEM in order to align areas of stable terrain. Each DEM of the pair was also co-registered to ICESat-2 bedrock elevations derived from ATL08 strong beam points^68^ acquired from OpenAltimetry^69^ to reveal co-registration offsets (indicating absolute error) between all three elevation sources^67^. During co-registration we correct slope- and aspect-dependent warping of elevation values following a standard procedure used by several large-scale glacier mass balance analyses^33,42^. Generally, warping is particularly prevalent in regions of high slope where small offsets in x and y generate large z offsets^70^.

### DEM differencing

To measure glacier surface elevation change across the five glaciers, the historical DEM (1968) was differenced from the modern reference DEM (2021) and a DEM from just prior to the LBIS collapse (2001). To determine the post-collapse component of the net 1968–2021 difference, the 2001 DEM was also differenced from the 2021 DEM. Voids in differenced DEMs were filled with interpolated values generated with Simple Kriging using up to 20 neighbours (Supplementary Material 4). The void-filled differenced DEMs were used for calculating mean glacier elevation changes^71^ over an areal extent that encompassed the main trunk and tributaries of each glacier. Glacier outlines were manually digitised by visual interpretation. The development of a floating ice tongue and extensive sea ice in 2021 obscured an obvious terminus position for Crane Glacier^24^, so the terminus for 2021 was picked at the first major lateral crevasse where downstream is predominantly fragmented ice. Boundaries in the upper catchment were defined at the uppermost ice cliffs which are distinct in slope data.

The mass balance of grounded ice was calculated using the difference in surface volume for the glacier area upstream of the grounding zone. Mass loss downstream of the grounding zone still contributes to sea-level rise but is difficult to quantify using surface elevation change on dynamic floating ice^72^. Volume change was calculated by multiplying the mean surface elevation change by the glacier surface area upstream of the grounding zone. MEaSUREs Antarctic grounding line positions for the year 1999 are the only available in this area^73^ and were used to define the glacier area for volume change calculations of the Jorum, Mapple, Melville and Flask Glaciers. Aberle et al.^46^ provide a better estimate for the Crane Glacier grounding line position for 2018, so we approximate this within ~ 500 m for volume calculations so that negligible error is induced from floating ice. Volume change was converted to mass units using an density of 917 kg m^-3^ to provide a first order estimate of water content. Mass loss was converted to sea-level rise equivalent using an ocean surface area^74^ of 3.625 × 10^6^ km^2^.

### Error assessment and vertical adjustments

**Figure 7:**
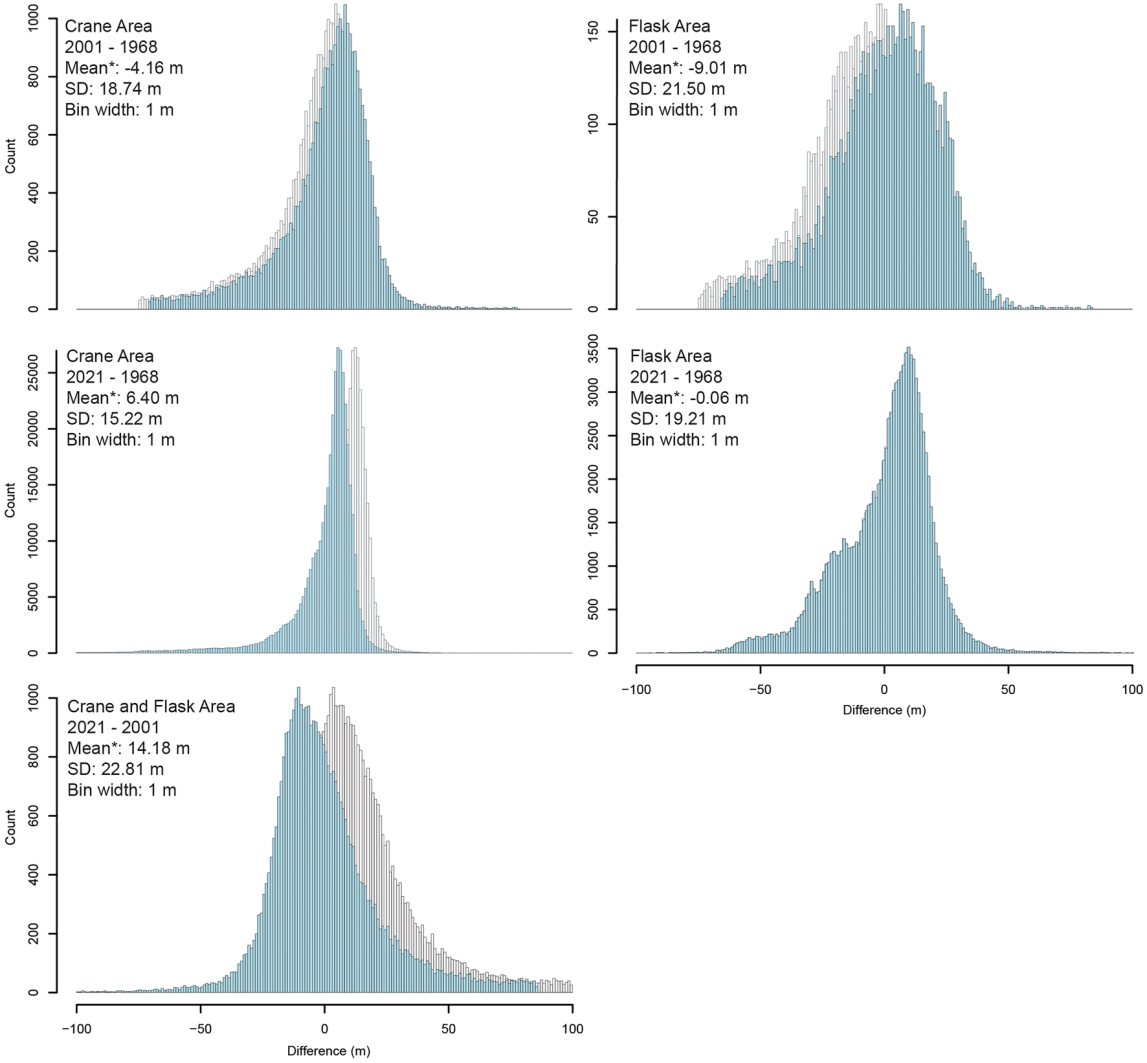
Histograms of difference values over stable terrain (bedrock where slope is < 30° and outliers > 3 SD are removed) between differenced DEM pairs. Values plotted in grey represent difference values after co-registration but before vertical offset adjustment (i.e., mean difference subtraction). Values plotted in blue represent difference values post-vertical adjustment. *Reported means are for pre-vertical adjustment values (grey).

For glacier change estimates, only the relative error between differenced DEMs is necessary to assess the uncertainty of the results^70^. The mean difference in ice-free areas between a pair of differenced DEMs represents systematic uncertainty^75^. For each differenced DEM, the mean difference of elevation values in all ice-free areas was calculated where slope is < 30° and outliers > 3 standard deviations were removed (Table 2). Typically, the mean offset is calculated for areas where slope is < 20°^76^ but in the steep-sloped mountains of the Antarctic Peninsula this condition limits almost all ice-free areas. The mean differences were universally subtracted from their respective DEMs^76,77^ before glacier change was calculated. Post-offset-adjustment, the uncertainty of individual points (standard deviation) was calculated (Table 2) and the variance illustrated with histograms of bedrock difference values (Fig. 7).

When characterising the uncertainty of a mean result, reporting the standard error accounts for the reduction in error that occurs when averaging a large sample size^78^. We use a standard error equation from Rolstad et al.^78^ for glaciers of larger spatial extent than the spatial autocorrelation range of the DEMs,

$${\sigma }_{\Delta \overline{h} }^{2}= {\sigma }_{\Delta z}^{2} \frac{1}{5} \frac{{A}_{cor}}{A},$$

where ${\sigma }_{\Delta \overline{h} }$ is the standard error of the mean elevation difference over glacier area *A*, ${\sigma }_{\Delta z}$ is the standard deviation of the elevation difference over stable terrain, and *A*_*cor*_ is the correlation area. We calculated spatial autocorrelation distances of 649 m to 2170 m over slope filtered, outlier-removed, stable terrain (Supplementary Material 5) and used this to determine correlation area for standard error calculations^76,77^. The error in volume change propagates from the standard error of the mean surface elevation change and the uncertainty in glacier area^33,42,70^,

$${\sigma }_{\Delta V}^{2}={\sigma }_{\Delta \overline{h} }^{2}+ {\sigma }_{A}^{2},$$

where ${\sigma }_{\Delta V}$ is the standard error of the volume change and ${\sigma }_{A}$ is the area uncertainty. The area uncertainty was calculated for each glacier by finding the area of a buffer around each glacier (with a width equal to the resolution used to define the glacier outline) as a percentage of the glacier area^42^. Glacio-isostatic adjustment and changes in firn air content may induce some uncertainty but the effects are small over this period compared to the total magnitude of change and are therefore ignored for the purposes of this work.

### Satellite image timeseries

All useful and available cloud-free, georectified satellite imagery in the pre-collapse period 1963–2001 and select scenes from after 2001 were acquired from USGS *EarthExplorer* archives (https://earthexplorer.usgs.gov/) and used to delineate ice margin change and validate surface elevation change results. The ice margin for a representative area along the main trunk of Crane Glacier, where the most dramatic change has occurred, was manually delineated using the high resolution 1968 orthophoto generated in this study. Changes in ice margins relative to 1968 were compared using the georectified satellite image timeseries 1963–2021. The earliest available optical image of the Larsen B area is a large-area declassified intelligence satellite image^79^ from 29th October 1963 which was georectified to the 1968 orthophoto with a 2nd order polynomial transformation using nine control points with a root mean square error of 37.3 m (Supplementary Material 1). The 1963 image has a pixel size of ~ 32 m but has poor dynamic range which limits the ability to resolve features in low contrast areas. Other pre-collapse satellite data include Landsat 4 and 7 optical band 3 images from 28th January 1989 and 23rd February 2000, respectively, and an ASTER optical band 3 nadir image from 22nd November 2001. Other ASTER optical band 3 nadir images were used to monitor ice margins post-ice shelf collapse and include scenes from 7th November 2002, 25th November 2006, and 14th February 2021. One Landsat 8 optical band 3 image was also analysed from 3rd December 2017. Complete metadata for images used in this study are listed in Supplementary Material 1.

### Supplementary Information

Supplementary Information 1.

Supplementary Information 2.

Supplementary Information 3.

Supplementary Information 4.

Supplementary Information 5.

## Data Availability

Digital elevation models and orthophotos produced from historical imagery are available to download and use from the Mendeley Data online repository: 10.17632/pfs54tfvym.1. Historical aerial imagery from 1968 is available at https://www.pgc.umn.edu/data/aerial/ and REMA DEMs are available at https://www.pgc.umn.edu/data/rema/. Historical satellite imagery from 1963, ASTER DEMs and satellite imagery, and Landsat 4, 7 and 8 satellite imagery are all available at via https://earthexplorer.usgs.gov/. MEaSUREs grounding line data is available at 10.5067/IKBWW4RYHF1Q. ICESat-2 data is available at https://nsidc.org/openaltimetry.
