# Supplementary material for: High-resolution elevation models of Larsen B glaciers extracted from 1960s imagery
Source: Sci Rep. 2024 Jul 8;14:14536. doi: 10.1038/s41598-024-65081-6 (PMC11231284; doi:10.1038/s41598-024-65081-6)
Supplement: Supplementary file 2 — Supplementary Information 2. [file 41598_2024_65081_MOESM2_ESM.pdf]

Supplementary Material 2 – Approximate camera positions, manual tie point positions, and GCP positions used in generation of the Crane Area and Flask DEM in Agisoft Metashape.

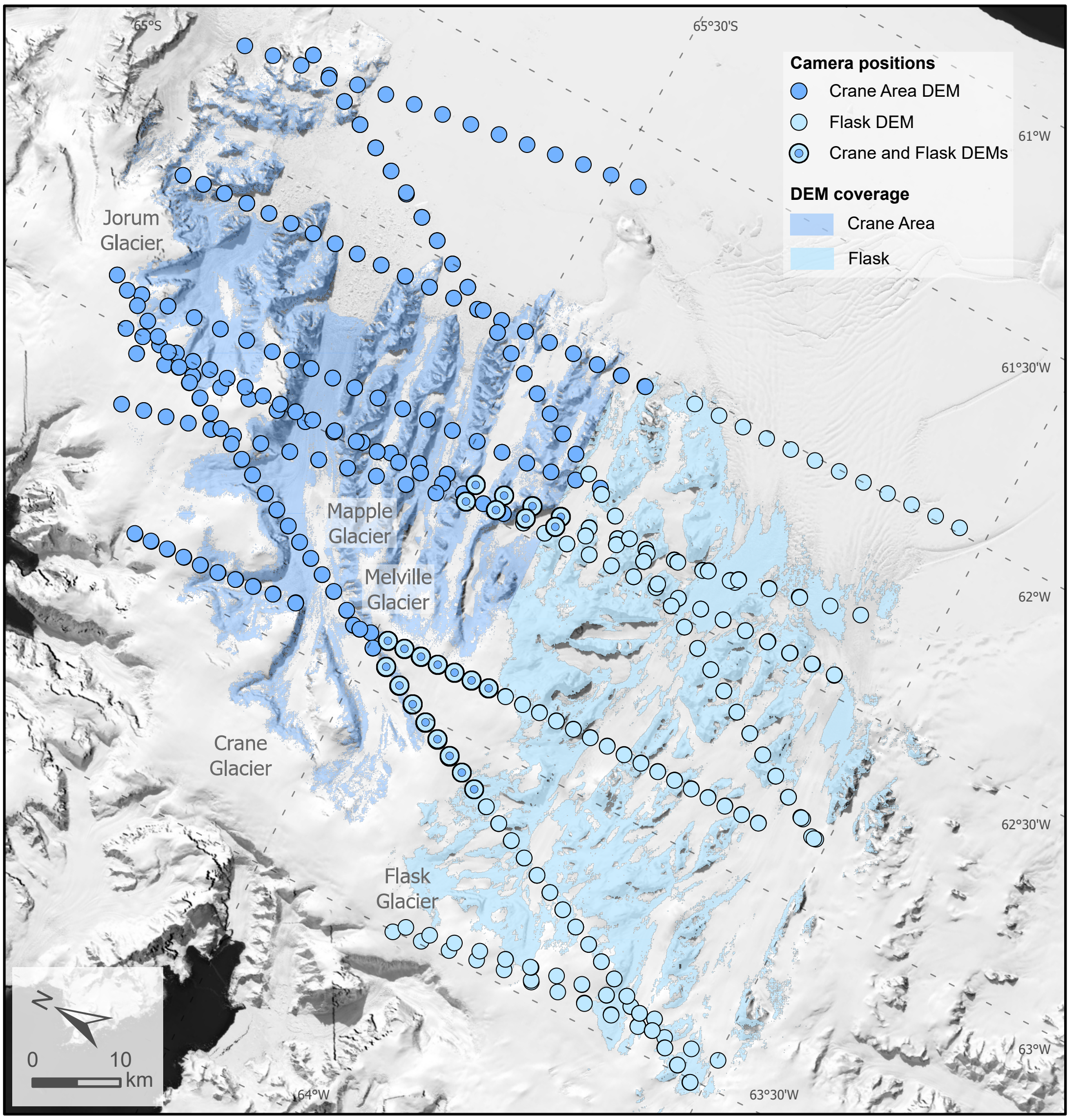

Basemap: Earthstar Geographic orthoimage, Projection: WGS84 Antarctic Polar Stereographic

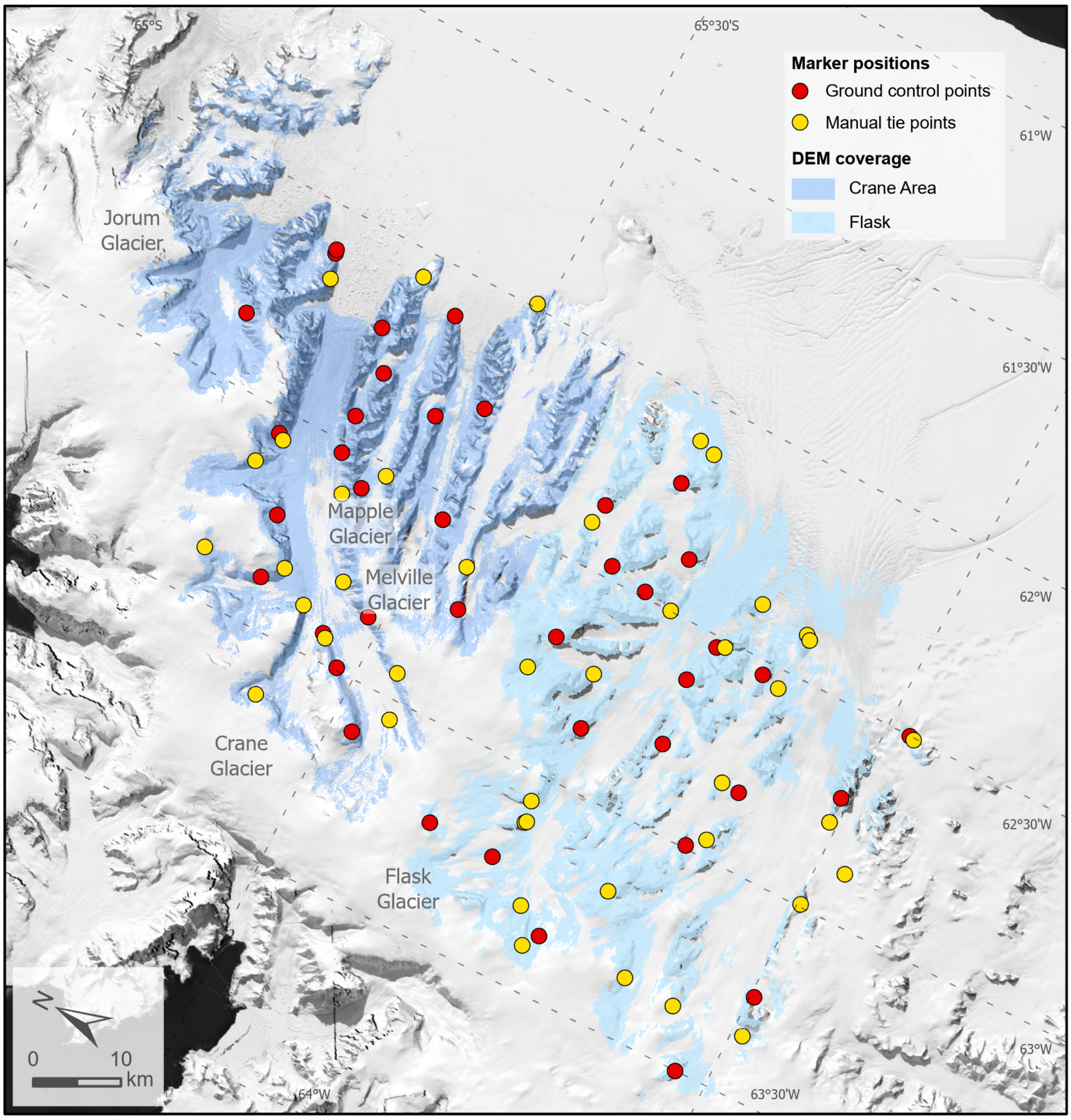

Basemap: Earthstar Geographic orthoimage, Projection: WGS84 Antarctic Polar Stereographic
