# Supplementary material for: High-resolution elevation models of Larsen B glaciers extracted from 1960s imagery
Source: Sci Rep. 2024 Jul 8;14:14536. doi: 10.1038/s41598-024-65081-6 (PMC11231284; doi:10.1038/s41598-024-65081-6)
Supplement: Supplementary file 4 — Supplementary Information 4. [file 41598_2024_65081_MOESM4_ESM.pdf]

Supplementary Material 4 - Areas of the differenced DEMs (1968 – 2021, 1968 – 2001 and 2001 – 2021) interpolated using Simple Kriging. Interpolated areas add negligible uncertainty since the Kriging prediction uncertainty is less than the standard deviation of elevation differences over stable terrain in all cases except in some areas on Crane (1968 – 2021, 2001 – 2021). In this case, elevation differences are underestimated in the areas of greatest error because interpolated points are being generated from points close to stable terrain (bedrock valley margins) where difference values are lower than on-glacier differences.

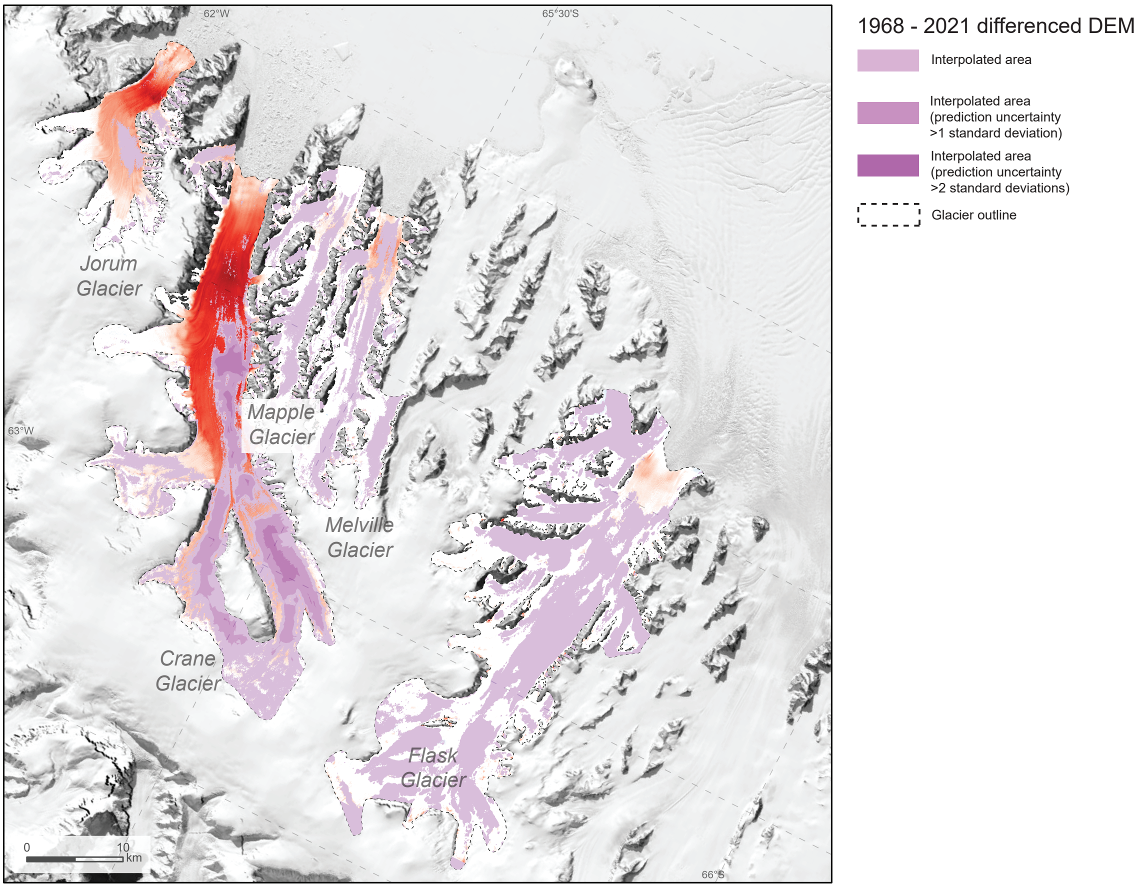


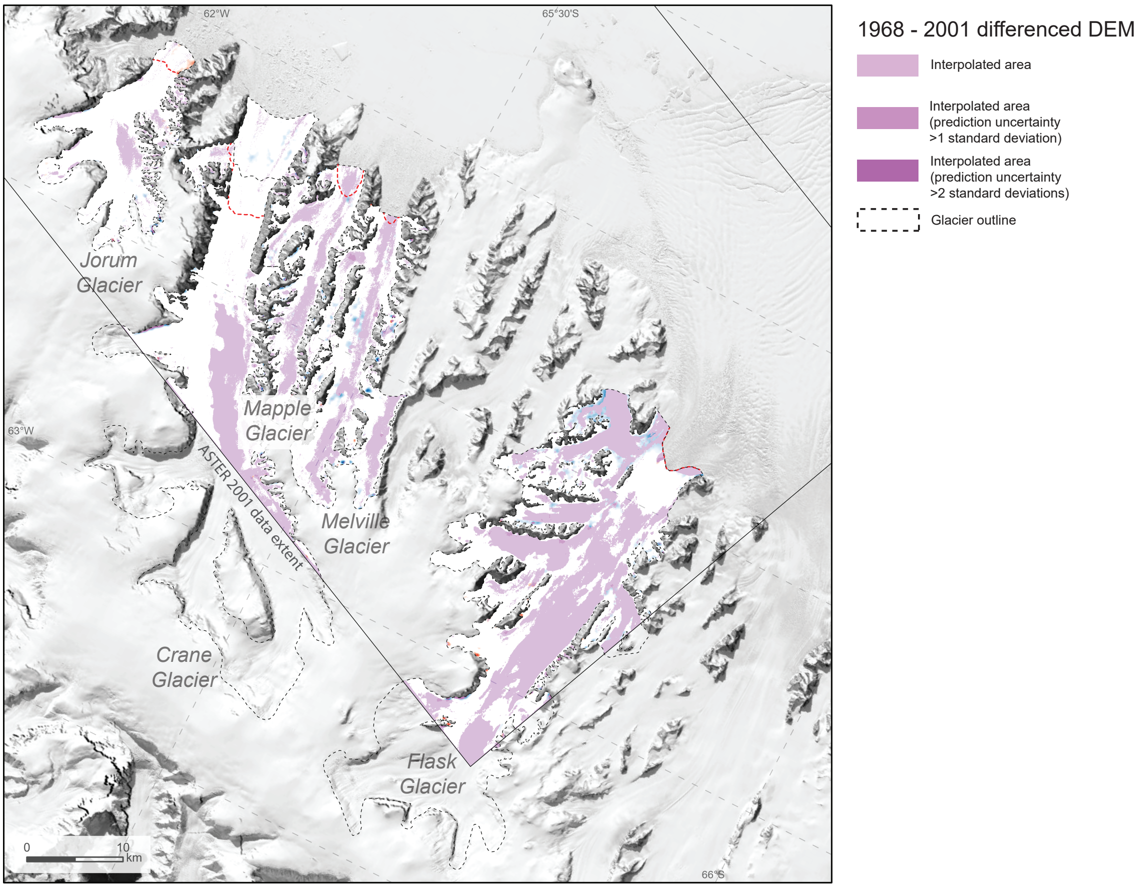

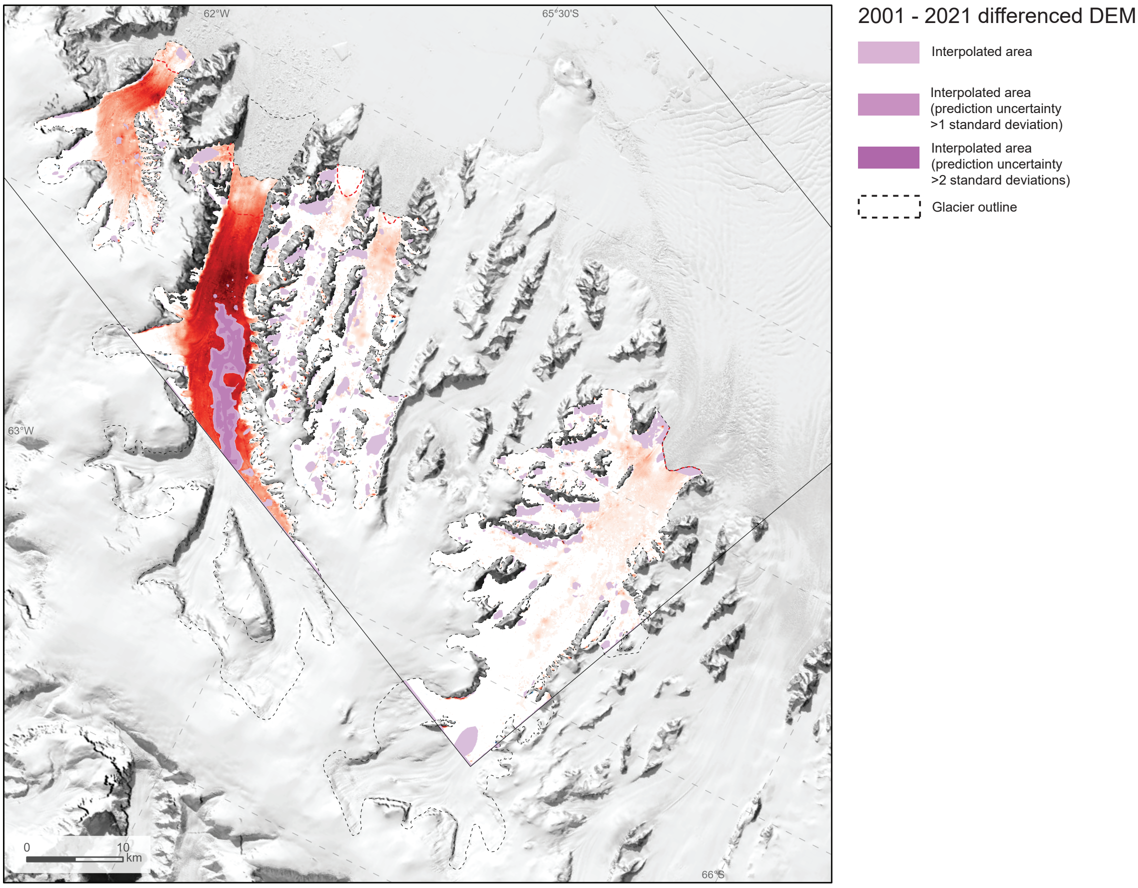
2001 - 2021 differenced DEM
Interpolated area
Interpolated area (prediction uncertainty >1 standard deviation)
Interpolated area (prediction uncertainty >2 standard deviations)
Glacier outline
62°W
65°30'S
63°W
66°S
0
10
km
